# Embryonal rhabdomyosarcoma of the adult prostate: case report and review

**DOI:** 10.1016/j.eucr.2021.101953

**Published:** 2021-11-22

**Authors:** Jeffrey Johnson, Aaron Brant, Christina Sze, Ananias Motta Burgos, Natallia Sheuka, Douglas Scherr

**Affiliations:** Weill Cornell Medical Center, 525 E 68th st, Suite Starr 900, New York, NY, 10065, USA

**Keywords:** Embryonal, Rhabdomyosarcoma, Adult, Treatment refractory

## Abstract

Herein is reported a case of embryonal rhabdomyosarcoma of the prostate in a 54-year-old male. The presenting symptoms were dysuria, hematuria, and systemic thrombotic events. Diagnosis was ascertained through a transurethral resection. The treatment course consisted of transurethral resection, prostatic embolization, chemotherapy with dactinomycin, vincristine, and cyclophosphamide, cystoprostatectomy, rectal excision, and external beam radiation. The patient succumbed to the fatality of this disease within six months of diagnosis. Rhabdomyosarcoma is a rare tumor that can arise in the prostate and this case highlights an unusually refractory and rapidly fatal case. Treatment guidelines are not established for adults with this disease.

## Highlights

• Appearance and course of embryonal rhabdomyosarcoma of the prostate.
• Refractory to medical, surgical, interventional radiologic, and radiation therapies.
• Strikingly poor prognosis and future applications for other cases.

## 1 Introduction

Rhabdomyosarcoma (RMS) is the most common type of soft tissue sarcoma in children, with the genitourinary system affected in 20% of cases.^1^ The presentation of embryonic rhabdomyosarcoma (ERMS) in adults however, is rare and represents <1% of all prostatic tumors.^2^ Treatment protocols are established for pediatric patients by the Intergroup Rhabdomyosarcoma Study Group, International Society of Pediatric Oncology Malignant Mesenchymal Tumor Group, and the RMS13 protocol from St. Jude's hospital among others. There are no established guidelines for adults with this disease, and prognosis is much poorer. Current literature demonstrates a median overall survival of approximately 29 months, with a 5-year overall survival of 31–42%.^3^, ^4^, ^5^

## 2 Case presentation

A 54-year-old male with a past medical history of hypertension, gout, and a mild ascending aortic aneurysm presented to an outside hospital for dysuria. His social history was noncontributory. His paternal grandfather had prostate cancer, and his maternal grandfather had lung cancer.

**Fig. 1:**
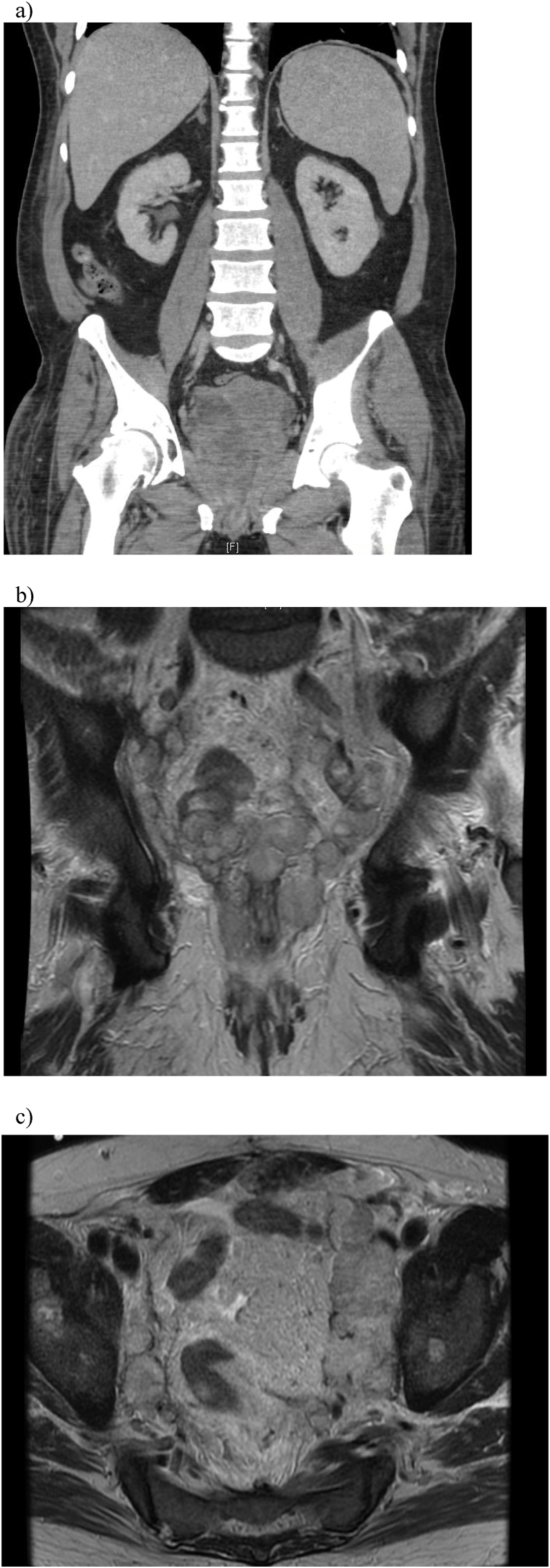
a) Initial CT abdomen/pelvis demonstrating a 776g prostate with highly unusual features. 1b, 1c) Coronal and axial view of MRI pelvis demonstrating recurrent tumor with invasion into the rectal stump and urethra prior to initiation of palliative radiation.

**Fig. 2:**
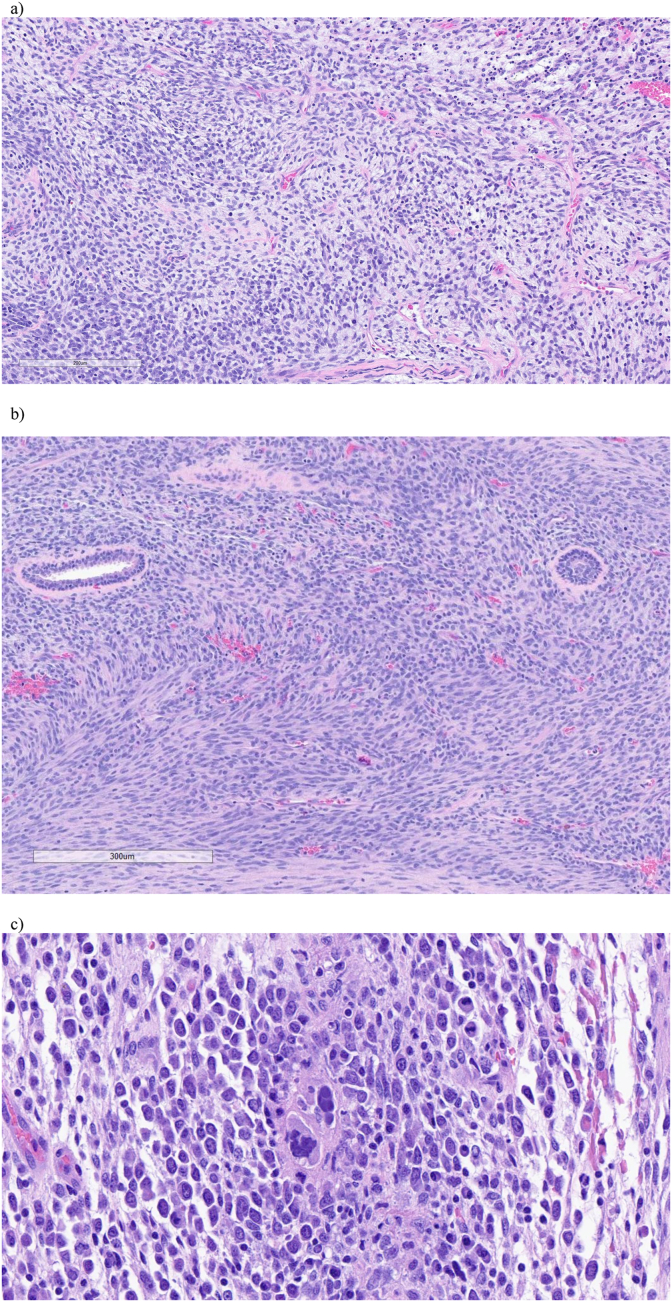
a, 2b) Embryonal rhabdomyosarcoma. Hematoxylin and eosin stain. A The area of tumor composed of primitive round cells in a myxoid background. B The spindle cell area of tumor infiltrating in between residual prostatic glands. 2c) Two tumor cells nuclei in the center of the image with hyperchromasia, multinucleation and significant enlargement relative to the background neoplastic cells. Hematoxylin and eosin stain, 40x magnification.

His symptoms were initially attributed to prostatitis, which was treated with a course of oral nitrofurantoin. Symptoms persisted, and he underwent subsequent urodynamics, which revealed normal bladder function. He developed gross hematuria after urodynamics, and underwent a CT urogram, demonstrating a 776g prostate with highly unusual features inconsistent with benign prostatic hyperplasia (Fig. 1). A cystoscopy and transurethral resection of the prostate was performed revealing a large and irregular tumor in the prostatic urethra. Pathology revealed embryonal rhabdomyosarcoma (ERMS) (Fig. 2a and b).

He developed shortness of breath and presented to the emergency department (ED) days after discharge, where he was diagnosed with a lower extremity deep vein thrombosis and pulmonary embolism. He was initiated on anticoagulation and had an inferior vena cava (IVC) filter placed. One week later, he returned to the ED with pain, gross hematuria, and clot retention. He was admitted and underwent a prostatic artery embolization. CT of the chest, abdomen, and pelvis revealed a 14.9 × 10 cm mass replacing the region of the prostate and seminal vesicles and invading the bladder with effacement of the fat plane between the rectum and the mass. MRI brain demonstrated no mass. The patient was discharged to initiate outpatient chemotherapy.

Vincristine, dactinomycin, and cyclophosphamide were started within a week after discharge from the hospital. While receiving chemotherapy, the patient presented to the hospital for weight loss, fatigue, pain, and gross hematuria with clot retention on two separate occasions. He underwent a cystoscopy with clot evacuation with supportive management of anemia including blood transfusions. After completion of three cycles of chemotherapy, the patient's symptoms of pain, weight loss, and hematuria persisted. Imaging of the abdomen and pelvis then demonstrated an increase in size of the mass to 17.4 × 9.5 cm with no discernible fat plane between the mass and the rectum, but no suggestion of rectal invasion and no lymphadenopathy. Given the progression of disease despite treatment, surgical management ensued.

The patient underwent a robotic-assisted cystoprostatectomy with Indiana pouch formation and pelvic lymphadenectomy (Fig. 2c). The tumor was adherent to the anterior rectal wall, so the rectum was excised and repaired, and an end ileostomy was created.

The immediate post-operative course was uneventful, and the patient was discharged on post-operative day (POD) 7. On POD 20, the patient was readmitted to the hospital for pelvic pain, chills, and fatigue and was discovered to have a perirectal bleed as well as a mass protruding from the urethra. His perirectal bleeding resolved after holding anticoagulation, and his urethral mass was resected again revealing ERMS. He received no chemotherapy in the interim during his recovery from surgery. An MRI pelvis was performed which demonstrated recurrent tumor with invasion into the rectal stump and urethra (Fig. 1b and c). There were also new left gluteus medius muscle lesions concerning for intramuscular metastasis and encasement with occlusion of the left internal iliac vein. CT chest demonstrated new bilateral lung nodules.

The patient was initiated on palliative external beam radiation. He received three treatment sessions of 2400 Gy each. 20 days after initiation of palliative radiation, the patient was admitted to the hospital for altered mental status, pain, and hypercalcemia. He was found to be hypoxic with increasing oxygen requirements and pulmonary edema. Acute renal failure ensued. He was treated with intravenous antibiotics of piperacillin-tazobactam for concern of urinary tract infection and pneumonia. Repeat CT imaging revealed progression of disease with new peritoneal metastases, malignant ascites, increased pelvic tumor size, increase in pulmonary nodules, and new bony metastases. He was started on renal replacement therapy. He developed gastric outlet obstruction. A family discussion yielded a decision for comfort care and the patient passed away 13 days after this admission.

## 3 Discussion

This case report outlines the progression of disease of an embryonal rhabdomyosarcoma of the prostate identified in a 54-year-old male. Current literature suggesting an overall survival median of 29 months (range 11.8–45.9 months) with embryonal subtype having the best prognosis of the RMS subtypes.^3^ This patient however, passed away 6 months from diagnosis. This tumor proved refractory to medical and surgical therapy. Although there were no signs of distant metastasis and no clear signs of local invasion at the time of diagnosis, this case progressed rapidly despite receiving dactinomycin, vincristine, and cyclophosphamide. The patient was to enroll in a clinical trial for renin-angiotensin system inhibitor after palliative radiation, however he passed away before enrollment.

## 4 Conclusion

This case is a reminder of the poor outcomes associated with adult ERMS. His disease proved uncharacteristically fatal, with little to no response to therapy. Extrapolated pediatric chemotherapy regimens, surgery, and radiation did not prove fruitful in this case. Optimizing outcomes in these patients is a work in progress, and the accumulation of cases like these can provide a greater body of evidence for future management.

## Consent

Informed consent for publication was obtained from this patient's surviving closest of kin, his wife.

## Funding

This research did not receive any specific grant from funding agencies in the public, commercial, or not-for-profit sectors.

## Declaration of competing interest

None.

## Abbreviations

ERMS: Embryonal rhabdomyosarcoma
RMS: Rhabdomyosarcoma
ED: Emergency Department

IVC Inferior Vena Cava.
